# Combined Cognitive-Psychological-Physical Intervention Induces Reorganization of Intrinsic Functional Brain Architecture in Older Adults

**DOI:** 10.1155/2015/713104

**Published:** 2015-02-24

**Authors:** Zhiwei Zheng, Xinyi Zhu, Shufei Yin, Baoxi Wang, Yanan Niu, Xin Huang, Rui Li, Juan Li

**Affiliations:** ^1^Center on Aging Psychology, Key Laboratory of Mental Health, Institute of Psychology, Chinese Academy of Sciences, Beijing 100101, China; ^2^University of Chinese Academy of Sciences, Beijing 100101, China; ^3^Magnetic Resonance Imaging Research Center, Institute of Psychology, Chinese Academy of Sciences, Beijing 100101, China

## Abstract

Mounting evidence suggests that enriched mental, physical, and socially stimulating activities are beneficial for counteracting age-related decreases in brain function and cognition in older adults. Here, we used functional magnetic resonance imaging (fMRI) to demonstrate the functional plasticity of brain activity in response to a combined cognitive-psychological-physical intervention and investigated the contribution of the intervention-related brain changes to individual performance in healthy older adults. The intervention was composed of a 6-week program of combined activities including cognitive training, Tai Chi exercise, and group counseling. The results showed improved cognitive performance and reorganized regional homogeneity of spontaneous fluctuations in the blood oxygen level-dependent (BOLD) signals in the superior and middle temporal gyri, and the posterior lobe of the cerebellum, in the participants who attended the intervention. Intriguingly, the intervention-induced changes in the coherence of local spontaneous activity correlated with the improvements in individual cognitive performance. Taken together with our previous findings of enhanced resting-state functional connectivity between the medial prefrontal cortex and medial temporal lobe regions following a combined intervention program in older adults, we conclude that the functional plasticity of the aging brain is a rather complex process, and an effective cognitive-psychological-physical intervention is helpful for maintaining a healthy brain and comprehensive cognition during old age.

## 1. Introduction

Normal aging is associated with cognitive decline in various domains, such as executive control, working memory, and episodic memory, and has been linked with structural morphology and functional changes in the brain [1, 2]. There has been accumulating evidence that older adults exhibit structural volumetric decreases, thinning of white matter tracts, and changes in functional activation patterns in several brain regions, specifically the prefrontal cortex (PFC) and medial temporal lobe (MTL) [3–7]. Fortunately, the aging brain exhibits experience-dependent plasticity, and increasing studies find that cognitive, physical, or social activity is greatly beneficial for the elderly to promote cognitive performance and optimize brain structure and function [8–10].

Neuroimaging studies have demonstrated that cognitive training can counteract age-related brain structural and functional losses. For instance, memory training can increase the cortical thickness [11] and induce greater activation in brain regions associated with self-initiated semantic strategy use [12] in healthy older adults. Moreover, memory training has been shown to enhance hippocampal activity during memory retrieval in patients with mild cognitive impairment (MCI) [13] and attenuate the differences in brain activation patterns between MCI patients and healthy controls [14]. Executive function training can induce functional alterations in cognitive control-related brain regions [15, 16]. Apart from cognitive training, physical exercise training has also been found to induce significant alterations in structural morphology and cerebral function [17–20].

Given that multiple factors and conditions have shown beneficial effects on the aging brain [2], it is expected that a combined cognitive-psychological-physical intervention including cognitive, physical, and social activities should be a more efficient approach to improve brain function in old age [21–23]. Following this assumption, a previous study from our group explored the effects of combined intervention including cognitive training, physical exercise, and group counseling on the functional plasticity in resting-state interregional connectivity in healthy older adults [24]. The results showed that the combined intervention enhanced the functional connectivity between the MTL and medial prefrontal cortex (mPFC), and the interregional connectivity changes were correlated with individual cognitive performance. This study confirmed that the combined intervention can improve the functional connectivity between the MTL and mPFC in the older adults. Nevertheless, these findings do not answer the question of whether or not the combined intervention could induce regionally brain functional plastic changes.

In recent years, the resting-state fMRI (RS-fMRI) has increasingly become a widely used method to investigate the brain functional plasticity [25]. RS-fMRI can be used to explore the intrinsic functional architecture of the brain without the need for participants to perform a specific task [26–28]. Previous studies usually use the resting-state functional connectivity (RSFC) analysis to examine the intervention effects on functional organization changes between distinct brain regions [24, 29]. However, the RSFC method can only be used to measure the interregional connectivity between spatially remote brain regions and strongly rests upon the regions of interest (ROI) defined with prior knowledge [30]. Regional homogeneity (ReHo) analysis is a unique RS-fMRI method for evaluating the local temporal synchronizations of spontaneous low frequency BOLD signals, which measures the similarity between the time series of a given voxel and its nearest neighbors [31]. It has been shown that the variations of ReHo values have neurobiological and structural basis [32]. As a data-driven approach, the ReHo method has high test-retest reliability [33, 34] and can detect regional changes that are induced by different conditions across the whole brain without requiring any prior knowledge [31]. It is widely applied in exploring brain function of healthy people and can predict individual performance in cognitive tasks [35–37]. Moreover, ReHo has been used to monitor disease progression in patients with Alzheimer's disease (AD) and MCI [38, 39]. Thus, the ReHo could be a potentially important complimentary method to examine the regional plasticity in aging brain.

In the present study, we aimed to further explore the regionally functional plasticity by using the ReHo method to do an exploratory analysis in the whole brain. The intervention group attended a combined intervention program consisting of cognitive training, physical exercise, and group counseling which has been introduced in Li et al. [24]. The cognitive training component focused on mnemonic and executive function training. Tai Chi, as a typical form of physical exercise, has been demonstrated to effectively improve the cognitive function [40], and optimize the regionally functional homogeneity of the intrinsic brain architecture in older adults [41]. In addition, group counseling was adopted to promote the psychological well-being of older adults, given that positive mood states could influence individual cognition and brain function [42]. We expected that this combined intervention program could alter the local functional homogeneity of the brain regions, as reflected by the changed ReHo values in the participants who attended the intervention, and we further speculated that these changes in local functional homogeneity would reflect improved individual cognitive performance.

## 2. Methods

In this study, healthy older adults' brain activity and neuropsychological performance were assessed before and after a 6-week combined cognitive-psychological-physical intervention. The effects of combined intervention on the functional plasticity in resting-state interregional connectivity have previously been reported for the participants in Li et al. [24]. This paper focused on the intervention effects on functional changes in the patterns of local spontaneous brain activity.

### 2.1. Participants

Forty-five healthy older adults were recruited from two communities near the Institute of Psychology, Chinese Academy of Sciences. After baseline evaluation, one community was randomly allocated to the intervention group (*n* = 26), and the other community formed the control group (*n* = 19). Participants were blind to the group allocation and study design. Participants were included in the study according to the same screen criteria used in Li et al. [24]. Of these, 11 participants were excluded from further analysis based on these criteria or for other reasons, and thus data for 34 participants were finally analyzed, with 17 in the intervention group (mean age: 68.59 years) and 17 in the control group (mean age: 71.65 years) [24].

The study protocol was approved by the Ethics Committee of the Institute of Psychology, Chinese Academy of Sciences. Written informed consent was obtained from all participants, and they were paid 200 Yuan for participation. The study was registered in the Chinese Clinical Trial Registry (ChiCTR) (http://www.chictr.org/): ChiCTR-PNRC-13003813.

### 2.2. Outcome Measures

A battery of neuropsychological tests was used to evaluate the intervention effects on cognitive function, health status, social support, and subjective well-being. The tests of cognitive function included the Montreal Cognitive Assessment-Beijing Version (MoCA-BJ) [43], Paired Associative Learning Test (PALT) [44], Digit Span Forward and Digit Span Backward [45], Trail Making Test (TMT) [46], Stroop Test [47], and Category Fluency Test (CFT) [48], which were used to assess the global cognition, episodic memory, working memory, executive function, and language ability, respectively. Health status was measured using the Medical Outcomes Study Short Form-36 (MOS SF-36) [49]. The level of social support was measured using the Social Support Rating Scale (SSRS) [50]. Subjective well-being was measured using the Satisfaction with Life Scale (SWLS) [51] and the Index of Well-Being (IWB) [52]. The test examiners were blind to the group allocation of participants (control or intervention).

### 2.3. Procedure

**Figure 1:**
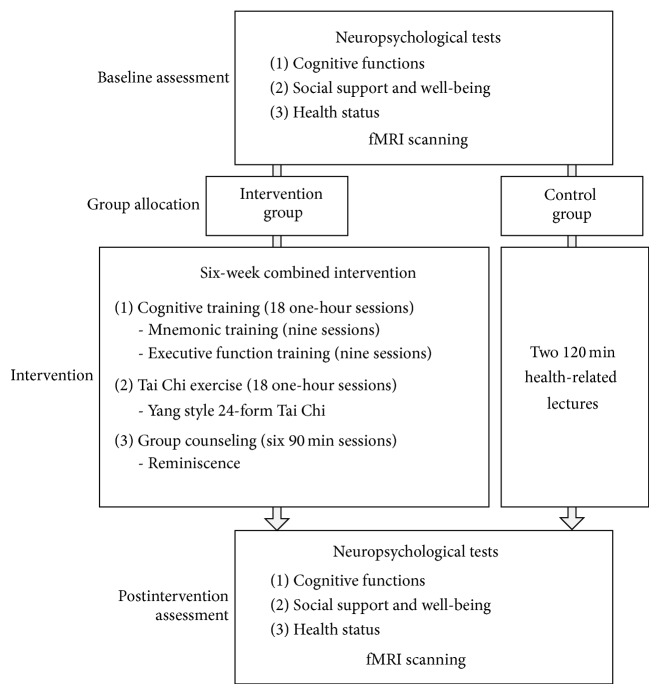
The procedure for this study.

Figure 1 displays the procedure for the study. All participants were subjected to a battery of neuropsychological tests and MRI scanning individually before and after the intervention. During the intervention process, the intervention group received a 6-week combined cognitive-psychological-physical intervention, including cognitive intervention, Tai Chi exercise, and group counseling, while the control group attended two 120 min lectures related to health and aging. The 18 one-hour cognitive training sessions were administrated three times per week and consisted of mnemonic training (MT; nine sessions) and executive function training (EFT; nine sessions). MT was designed to teach older adults elaborate encoding and retrieval strategies, such as interactive imagery, sentence generation, and the method of loci. EFT was designed to train older adults three components of executive function: inhibition, switching, and updating. The 18 one-hour physical exercise sessions required participants to learn the Yang Style 24-form Tai Chi three times per week. Group counseling sessions aimed at promoting the psychological well-being of older adults through reminiscence and were conducted as six weekly sessions of 90 min each. Please refer to Li et al. [24] for details about the intervention program.

### 2.4. Statistical Analysis of Neuropsychological Data

The demographic and clinical characteristics of participants in both intervention and control groups were examined by using chi-square, *t*, or nonparametric (Mann-Whitney) tests. The repeated measures two-way analysis of variance (ANOVA) with the within-subject factor of intervention (pre, post) and the between-subject factor of group (control, trained) was conducted on the performance for each test to examine the intervention effect. All statistical analyses were conducted using SPSS 19.0 (IBM Corporation, Somers, NY).

### 2.5. Image Data Acquisition

All images were collected using a 3-Tesla Siemens Trio scanner (Erlangen, Germany) at the Beijing MRI Center for Brain Research. Each participant was scanned before and after the intervention using the same fMRI protocols. During each scan, participants lay supine with head snugly fixed by a belt and foam pads to minimize head motion. They were instructed to lie quietly, keep their eyes closed, and not think of anything in particular. For each participant, resting-state fMRI data was acquired using an echo-planar imaging (EPI) sequence with the following parameters: time repetition (TR) = 2000 ms; time echo (TE) = 30 ms; flip angle = 90°; field of view (FOV) = 200 × 200 mm^2^; thickness = 3.0 mm; gap = 0.6 mm; acquisition matrix = 64 × 64; in-plane resolution = 3.125 × 3.125; 33 axial slices; 200 volumes. A high-resolution, three-dimensional T1-weighted structural image was also collected for each subject with the following sequence: 176 slices; acquisition matrix = 256 × 256; voxel size = 1 × 1 × 1 mm³; TR = 1900 ms; TE = 2.2 ms; flip angle = 9°.

### 2.6. Image Processing and Analysis

Image processing was performed using Statistical Parametric Mapping (SPM8, http://www.fil.ion.ucl.ac.uk/spm/) and Data Processing Assistant for Resting-State fMRI (DPARSF) V2.0 Basic Edition [53].

The first five images for each subject were discarded to allow for equilibration of the magnetic field and the acclimatization to the scanning environment. The 195 remaining images were first corrected for intravolume acquisition time differences between slices and intervolume geometrical displacement due to head motion. Participants included in this study were restricted to head motion of less than 2.0 mm in any direction and 2.0° of angular motion during the resting-state scan. The functional images were then normalized to the standard space of the Montreal Neurological Institute (MNI) and resampled to a voxel size of 3 × 3 × 3 mm³. Following this, detrending and temporal band pass filtering (0.01–0.08 Hz) of the fMRI data was carried out to reduce the effects of low-frequency drift and physiological high-frequency noise.

Following Zang et al. [31], the ReHo value in the brain was measured using Kendall's coefficient of concordance (KCC) between the time series of a given voxel and its nearest 26 neighbors. Specifically, we first calculated the KCC for each voxel across the whole brain to derive the ReHo map for each subject. For standardization purposes, each ReHo map was then divided by the mean ReHo value of the entire brain. Finally the ReHo maps were spatially smoothed with a 4 mm full-width at half-maximum (FWHM) Gaussian kernel.

For statistical analysis, we first used a one-sample *t*-test to compare the ReHo maps for intervention and control groups both before and after intervention and then performed a whole-brain voxel-wise Group (control, trained) × Intervention (pre, post) ANOVA on the ReHo maps to detect regions showing intervention-related changes. Clusters were considered significant at a level of *P* < 0.01 for the combined voxel-extend threshold of an uncorrected voxel and cluster extent >486 mm^3^, as determined using the Monte Carlo simulation with AlphaSim correction to *P* < 0.01. In further studies, the regions showing a significant Group × Intervention interaction were defined as regions of interest (ROIs). We extracted the mean ReHo value in each ROI and used paired sample *t*-tests (*P* < 0.05) to examine the effects of intervention on regional ReHo in each of the ROIs for the two groups.

Finally, to examine whether intervention-related changes in brain activity are associated with improvements in cognition, the correlations between intervention-related ReHo changes in the ROIs and changes in cognition variables were investigated (*P* < 0.05, Bonferroni corrected). Between-group comparisons were conducted with Fisher's *r* to *Z* transformation to directly compare two correlation coefficients.

## 3. Results

### 3.1. Demographic and Clinical Characteristics

Table 1 displays the demographic and clinical characteristics of the participants in the intervention and control groups. The two groups did not differ significantly in age, years of education, gender, or on the MoCA-BJ, Center for Epidemiologic Depression Scale (CES-D) [54], and ADL (Activities of Daily Living) scores [55].

### 3.2. Effects of Combined Cognitive-Psychological-Physical Intervention on Behavioral Performance

ANOVA analyses revealed that, after the intervention, significant improvements in the PALT and vitality (VT, a dimension of MOS SF-36) and greater improvements in SSRS were found in the intervention group, but no significant or smaller improvements in these tests were found in the control group. In addition, the results also showed that the performance on TMT and SWLS did not change for the intervention group but decreased for the control group after the intervention (refer to Li et al. [24] for detailed behavioral results).

### 3.3. ReHo Maps and the Intervention-Related Changes

**Figure 2:**
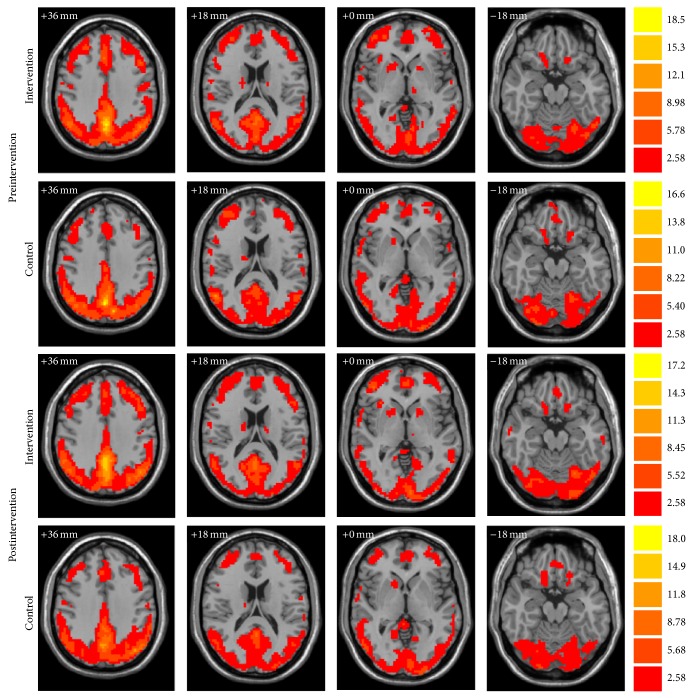
Whole-brain analyses of ReHo are visualized, respectively, for intervention and control groups both before and after intervention. Bars at the right show *t*-values. Following radiological convention, the left side of the image corresponds to the right side of the subject.

**Figure 3:**
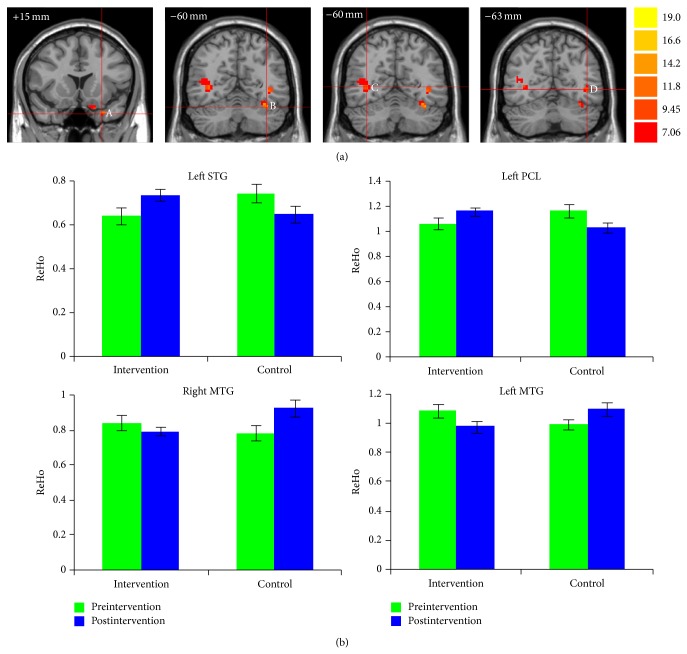
Combined cognitive-psychological-physical intervention altered the ReHo of spontaneous brain activity in the left STG, left PCL, and bilateral MTG. (a) Coronal view of brain regions showing significant Group × Intervention interactions in ReHo for (A) left STG; (B) left PCL; (C) right MTG; (D) left MTG. The numbers above each image refer to the *y* plane coordinates of MNI. Left in picture is right in the brain. (b) Bar plots showing the mean ReHo of spontaneous brain activity in these ROIs before and after intervention, for the intervention and control groups.

Figure 2 demonstrates the ReHo maps before and after intervention for both groups. The ReHo maps displayed very similar spatial patterns to the default-mode network. The whole-brain voxel-wise Group × Intervention ANOVA analysis showed that there were four regions with significant interactions for Group × Intervention (AlphaSim correction, *P* < 0.01), including the left superior temporal gyrus (STG; peak MNI location: −27, 15, −33; 23 voxels); left posterior lobe of cerebellum (PCL; peak MNI location: −36, −60, −24; 22 voxels); left middle temporal gyrus (MTG; peak MNI location: −42, −63, 0; 27 voxels); and right MTG (peak MNI location: 36, −60, 3; 73 voxels) (Figure 3(a)). The paired *t*-tests of the mean ReHo for spontaneous brain activity in each of the four regions of interests (ROIs) demonstrated that combined intervention significantly increased the ReHo in the left STG (*P* = 0.019) and left PCL (*P* = 0.038) but decreased the ReHo in the left MTG (*P* = 0.032) in the intervention group. By contrast, the control group showed significantly decreased ReHo in the left STG (*P* = 0.005) and PCL (*P* = 0.009) and increased ReHo in the left MTG (*P* = 0.068) and right MTG (*P* < 0.001) (Figure 3(b)).

### 3.4. Correlations between Intervention-Related Changes in Brain Activity and Cognitive Performance

**Figure 4:**
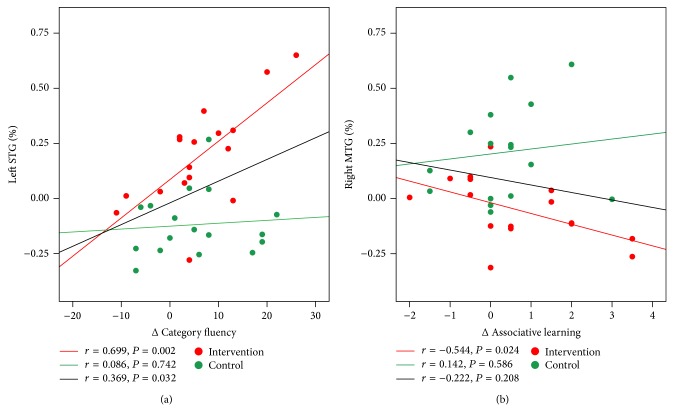
Correlations between the intervention-related changes in ReHo of spontaneous brain activity and changes in cognitive performance for all participants. Data of the intervention group are colored in red and those of the controls are colored in green. The red fit line on each graph represents the relationship between the intervention-related changes in ReHo and cognitive performance in the intervention group, and the green fit line represents the relationship in the control group. The black fit line displays the relationship between the intervention-related changes in ReHo and cognitive performance for all participants from the two groups.

Correlations were calculated between the ReHo measures of four ROIs and neuropsychological test scores on cognition (TMT, PALT, and CFT). We performed the correlations analyses between the intervention-related changes in ReHo of spontaneous brain activity ((postintervention − preintervention)/preintervention) and changes in cognitive measures (postintervention − preintervention). In the intervention group, the results showed that intervention-related ReHo changes in the left STG were significantly positively correlated with changes in the CFT in the participants who attended the intervention activities (*r* = 0.699, *P* = 0.002) at a Bonferroni correction threshold of 0.0042 (0.05/12). On the other hand, no significant correlation between the ReHo changes in the left STG and gains of the CFT was found in the control group, *r* = 0.086, *P* = 0.742 (Figure 4(a)). A further analysis directly comparing the correlations between the two groups revealed that the two correlation coefficients were significantly different from one another, Fisher's *r* to *Z* = 2.06, *P* = 0.039. When a more liberal threshold of *P* < 0.05 without correction was applied, we found that the intervention-related ReHo changes in the right MTG were negatively correlated with the intervention-related increase in the total PALT scores in the intervention group, *r* = −0.544, *P* = 0.024. In the control group, no significant correlation was observed between the ReHo changes in the right MTG and gains of the PALT, *r* = 0.142, *P* = 0.586 (Figure 4(b)). Subsequent analysis revealed that there was significant difference between the two correlation coefficients, Fisher's *r* to *Z* = −1.99, *P* = 0.047.

## 4. Discussion

We explored the effects of a combined cognitive-psychological-physical intervention on brain functional plasticity in healthy older adults using RS-fMRI. The results revealed improved cognitive performance and alterations in the ReHo of local spontaneous brain activity in the superior and middle temporal gyri and the posterior lobe of the cerebellum for the intervention group. These intervention-related ReHo changes were correlated with individual improvements in cognitive performance. The present study provides evidence that combined cognitive-psychological-physical intervention can induce functional plastic changes in the lateral temporal lobe and cerebellum in healthy older adults.

Based on the whole-brain voxel-wise analysis, the fMRI results revealed that combined intervention significantly altered the coherence of local spontaneous brain activity in the left superior temporal gyrus (STG) and middle temporal gyrus (MTG) in the intervention group. Although previous studies have primarily emphasized the effects of aging on the frontal cortex and MTL, many studies have implied that the lateral temporal lobe is also vulnerable. Decreases in the density of gray matter in the left superior temporal region have been reported [56] and the bilateral temporal cortices show obvious atrophy [57, 58] and decreased metabolism [59] in the elderly. The left STG has previously been thought to play an important role in speech comprehension; however, this region is also involved in speech production [60]. Studies of conduction aphasia where there is damage in the left STG have confirmed the role of the STG in speech production, by revealing good comprehension but phonemic paraphasias and naming difficulties [61]. A recent study has demonstrated plasticity in this brain region [62]. The researchers found that the cortical thickness of the left STG increased in those who participate in foreign language training, and this structural change was positively correlated with posttest proficiency in language use.

It is well known that spontaneous resting state brain activity may serve an important role in brain function [27]. Furthermore, previous studies have proved that ReHo measures are associated with individual differences in cognitive performance [35–37]. Consequently, it is reasonable to speculate that the present finding of enhanced coherence of local spontaneous brain activity in the left STG for the intervention group suggests that combined cognitive-psychological-physical intervention can induce brain functional plasticity in the superior temporal cortex and lead to functional improvements in this brain region associated with speech production.

Correlation analysis further supports this proposal by showing a positive correlation between intervention-related changes in coherence in the left STG and changes in the CFT for the intervention group. The CFT is a typical neuropsychological test used to examine speech production, and performance of the CFT declines with normal aging [63, 64]. The CFT greatly depends upon the function of the temporal lobe [65], and patients with semantic dementia and temporal lobe atrophy show poor category fluency [66, 67]. The results of an fMRI study revealed greater activation in the left STG during the category fluency tasks, further confirming the important role of this region in language production [68]. In the current study, our correlation results are consistent with an important functional role for the left STG in CFT performance and provide evidence that the patterns of local spontaneous resting brain activity in the left STG could reflect individual CFT performance in older adults.

Interestingly, the present results showed a concomitant increase and decrease in the ReHo of spontaneous brain activity in the left STG and left MTG for the intervention group. Although the exact reasons are still unclear, we believe that the present results to some extent echo heterogeneous functional characteristics in subregions of the lateral temporal lobe. The MTG is thought to be involved in mapping between the phonological forms of words and their meanings and serves as a sound-to-meaning interface [60, 69, 70], and meta-analyses of neuroimaging literature have confirmed the importance of its role in semantic processing [71, 72]. The MTG has extensive structural and functional connectivity with frontal, parietal, and temporal regions in the resting brain, and this is thought to play a central role in language comprehension [73]. Therefore, the present finding of decreased coherence of local spontaneous resting activity in left MTG suggests functional plastic changes in the middle temporal cortex and may reflect greater efficiency of information processing in the intervention group for this brain region involved in language processing.

Although the ReHo of the right MTG did not change significantly after intervention in the intervention group, within a more liberal threshold (*P* < 0.05), our results showed a significant correlation between coherence changes in the right MTG and the PALT for the intervention group. The PALT is a neuropsychological test used to examine the episodic memory using word pairs [44]. Previous task-based fMRI studies have found that activity of the right MTG during encoding is correlated with subsequent memory performance, suggesting that brain activity in regions in charge of language comprehension is predictive of episodic memory with narrative materials [74, 75]. In the present study, our results further confirm the important role of the MTG in the processing of language materials and provide evidence that intervention-induced changes in patterns of local spontaneous brain activity in the right MTG may also predict individual episodic memory in older adults.

The combined cognitive-psychological-physical intervention also induced enhanced coherence of local spontaneous activity in the left posterior lobe of the cerebellum (PCL) for the intervention group. The traditional views are that the cerebellum is mainly responsible for motor coordination and motor learning, but in recent years, investigators have paid more attention to the higher-order functions of the cerebellum, such as working memory, executive functions, and emotional control [76–78]. Damage to the cerebellum, especially in the lateral hemisphere of the posterior cerebellum, will induce cerebellar cognitive affective syndromes (CCAS), which are characterized by impairments in executive, visual-spatial, linguistic abilities, and affective disorders [79]. Functional topography studies within the cerebellum have further indicated that anterior portions of the cerebellum mainly support motor function, whereas the posterior regions of the cerebellum are mainly involved in cognitive and emotional processing [80–82].

A substantial body of evidence has demonstrated that the cerebellum also shows age-related decreases in structural morphology and function [83]. For instance, a recent study found age-related decreases in resting-state cerebello-cortical functional connectivity, and lower connectivity was associated with poorer cognitive performance in older adults [84]. Our intervention program contains cognitive training (mnemonic and executive function training) and group counseling, so the present findings of increased local coherence of spontaneous brain activity in the PCL suggest that intervention-induced functional optimization of this region contributes to higher levels of cognitive processing and emotional control in the intervention group.

Taken together, the present study confirms that the combined cognitive-psychological-physical intervention induces regionally brain functional reorganization. However, it should be noted that we cannot ascertain the exact contribution of each specific training component to the brain functional plasticity. Interestingly, though our previous study observed enhanced resting-state functional connectivity between the MTL and PFC [24], the present findings did not show significant intervention-induced changes of ReHo values in these two brain regions. These results indicated that the functional plasticity of the aging brain is a complex process. Further studies are required to elucidate the underlying causes and mechanism. In addition, we observed both a positive correlation in the left STG and a negative correlation in the right MTG between intervention-related changes in ReHo and cognitive performance in the present study. The relationship linking changes of ReHo values and individual behavioral performance is extremely complicated. The higher ReHo level does not necessarily result in better performance and vice versa. Evidences can be found in previous correlation studies between ReHo and cognitive function [35, 39]. The underlying meaning of increased or reduced ReHo values remains unclear. It seems that both positive and negative correlations could make the inference that the changes of ReHo may reflect individual behavioral performance.

Several limitations should be noted. Firstly, the control group only attended two 120 min lectures and is therefore not a completely active control group. Nevertheless, as noted in Takeuchi et al. [85], the lack of an active control group is actually a commonly used approach in neuroimaging studies involving training. The positive effects induced by intervention cannot simply be attributed to active stimulation [86]. In the present study, the intervention group showed alterations in patterns of local spontaneous brain activity in the brain regions that are vulnerable to aging, and moreover, the ReHo changes were correlated with the gains of individual cognitive performance. We believe that these beneficial effects at least partially reflect the function of the combined intervention. Secondly, it is unfortunate that the participants could not be randomized to the intervention and control groups. This was necessary because the participants were enrolled from two communities, and if there were participants from the same community in each group, it would be possible for them to communicate the intervention contents and thus confound the intervention effects. Thirdly, we found that combined cognitive-psychological-physical intervention could improve the functional organization of the resting brain, and although it could be supposed that this corresponds to an overall increase in cellular activity and metabolic rate in the relevant brain regions [86], the underlying mechanisms for this functional plasticity are still obscure. Lastly, we did not assess the long-term effects of the combined intervention. Future research is needed to determine whether, and to what extent, these beneficial effects can be maintained over time.

## 5. Conclusion

In summary, the present study extended our previous findings by showing that combined intervention could optimize the intrinsic functional brain architecture in the temporal cortex and cerebellum in the normal elderly. Moreover, the changes in ReHo of local spontaneous resting state activity could predict improvements in individual cognitive performance. These results further suggest the effectiveness of the intervention in curbing the loss of brain function in older adults.

## Figures and Tables

**Table 1:** Demographic and clinical characteristic of the participants (mean and standard deviations).

	Intervention group (*n* = 17)	Control group (*n* = 17)	*P* value
Age (years)	68.59 (5.65)	71.65 (4.00)	0.08^a^
Gender (male/female)	9/8	11/6	0.50^b^
Education (years)	13.29 (3.06)	14.65 (3.26)	0.22^a^
MoCA	26.29 (2.69)	25.18 (2.53)	0.33^c^
CESD	7.00 (5.53)	6.82 (6.06)	0.85^c^
ADL	14.12 (0.49)	14.18 (0.73)	0.97^c^

Note: ^a^The *P* value was obtained using a two-sample two-tailed *t* test. ^b^The *P* value was obtained using a two-tailed Pearson chi-square test. ^c^The *P* value was obtained using nonparametric (Mann-Whitney) test.
